# Structural Motifs as Programmable Design Parameters for Tuning Vascular Mechanics in Fiber-Reinforced Hydrogel Grafts

**DOI:** 10.3390/biomimetics11080591

**Published:** 2026-08-19

**Authors:** Dekel Maroz, Adi Aharonov, Hod Hoenig, Mirit Sharabi

**Affiliations:** Department of Mechanical Engineering and Mechatronics, Ariel University, Ariel 407000, Israel; dekelm@ariel.ac.il (D.M.); adiben@ariel.ac.il (A.A.); hod.baranes@msmail.ariel.ac.il (H.H.)

**Keywords:** vascular grafts, biomimetic design, structural motifs, fiber-reinforced hydrogels, nonlinear mechanics, compliance matching, soft composites

## Abstract

Replicating the nonlinear, pressure-dependent mechanical behavior of native arteries remains a central challenge in small-diameter vascular graft design, where compliance mismatch between synthetic grafts and host vessels is strongly linked to graft failure. Building on a silk fiber-reinforced alginate–polyacrylamide interpenetrating polymer network (IPN) hydrogel platform, we investigated whether biomimetic vascular structural motifs, specifically fiber reorientation and crimp, can be used as programmable design parameters to tune the tensile and pressure-dependent mechanical response of tubular constructs toward native coronary artery behavior. Three architectures were fabricated: a cross-plied (CP) baseline, a 25° reoriented configuration, and a crimped CP configuration. Under internal pressurization, fiber reorientation and crimp significantly increased compliance at low physiological pressures, with a consistent directional trend across the full pressure range, by extending the low-stiffness toe region preceding fiber recruitment while preserving tensile stiffness. Across the investigated pressure range, all architectures exhibited compliance within the reported range of native coronary arteries, with crimped constructs producing pressure–diameter behavior that most closely resembled young coronary arteries, whereas the CP baseline more closely resembled aged coronary arteries. These findings demonstrate that biomimetic structural motifs, without altering material composition, can serve as programmable design parameters for engineering vascular mechanics, enabling a single material platform to reproduce distinct physiological mechanical phenotypes through architecture alone.

## 1. Introduction

Small-diameter vascular grafts remain a major challenge in cardiovascular replacements [1,2], with long-term failure often associated with a mechanical mismatch between the graft and the native vessel [3,4,5,6]. Despite decades of development, currently available synthetic grafts exhibit poor patency rates in small-diameter applications (<6 mm), primarily due to thrombosis and intimal hyperplasia [1,2,7]. Among the mechanical factors implicated in graft failure, compliance mismatch is one of the most critical determinants of graft performance and long-term patency [3,5,6,8].

Vascular compliance describes the relationship between pressure and diameter, directly affecting hemodynamic conditions, wall stresses, and the interaction between the graft and surrounding host tissue [4,8,9]. Native arteries exhibit a nonlinear pressure–diameter response, characterized by high compliance at low pressures and progressive stiffening at higher pressures [9,10,11]. This behavior is further emphasized by aging, with aged coronary arteries exhibiting reduced compliance and earlier stiffening compared to younger vessels [12,13,14,15]. These age-dependent differences emphasize that the mechanical target for a vascular graft is not a single universal arterial response, but rather a physiological range that may vary with the patient and vascular condition [12,14,15].

In native arteries, nonlinear mechanics arise from the progressive recruitment of collagen fibers embedded within an elastin-rich matrix [11,16]. At low pressures, elastin dominates the response, whereas increasing pressure gradually straightens initially crimped collagen fibers, producing the characteristic J-shaped stiffening behavior [17,18,19,20,21]. Consequently, fiber orientation, crimp, and recruitment govern pressure-dependent arterial mechanics, while age-related reductions in crimp and earlier fiber recruitment contribute to increased arterial stiffness [14,15].

Compliance mismatch between vascular grafts and native arteries has been strongly associated with adverse biological and mechanical consequences, including disturbed flow patterns, altered wall shear stress, and the development of intimal hyperplasia at the anastomosis [3,4,5,8]. These effects are closely linked to differences in deformation behavior under pulsatile loading, where overly stiff grafts fail to expand synchronously with the host vessel [3,4,6,8]. Accordingly, reproducing the native pressure-dependent mechanical response across the physiological pressure range remains a key requirement for successful small-diameter vascular grafts [6,8,9].

Conventional synthetic grafts, such as expanded polytetrafluoroethylene (ePTFE) and polyethylene terephthalate (Dacron), typically exhibit limited compliance and fail to reproduce the nonlinear mechanical response of native arteries [1,7,9,17]. Hydrogel-based systems have emerged as promising candidates due to their high water content and tunable bulk properties [22,23], but generally lack the structural organization required to replicate the anisotropic, nonlinear, and recruitment-driven behavior of vascular tissues [9,18,23]. Alternative approaches, including electrospun nanofibrous grafts [24,25], decellularized natural conduits [26], and acellular tissue-engineered vessels [27,28], have further advanced the field, with several achieving clinically relevant mechanical performance and one recently receiving regulatory approval for vascular trauma repair [28].

Fiber-reinforced hydrogels have emerged as a promising biomimetic platform for engineering vascular mechanics through controlled architectural design [29,30,31,32,33,34,35,36,37]. By combining reinforcing fibers with a compliant matrix, these materials partially reproduce the hierarchical organization and anisotropic mechanical behavior of native soft tissues. Previous studies have shown that architectural parameters such as fiber orientation can systematically influence stiffness, strength, and stretchability [29,30,33,34,38]. However, vascular grafts are typically designed and evaluated by optimizing individual mechanical benchmarks, such as burst pressure or compliance over a limited pressure range, rather than by translating the structural principles that govern native pressure-dependent mechanical behavior [24,25,26,27,28]. Consequently, it remains unclear whether vascular structural motifs themselves can serve as programmable engineering design parameters for systematically controlling the nonlinear pressure–diameter response of synthetic vascular grafts. Addressing this challenge requires biomimetic platforms in which pressure-dependent mechanical behavior is programmed through structural architecture rather than optimized solely through material composition.

Among these systems, fiber-reinforced interpenetrating polymer network (IPN) hydrogels provide a versatile platform for engineering biomimetic vascular mechanics. These composites combine a compliant IPN matrix with continuous reinforcing fibers, providing enhanced toughness, stretchability, and tunable anisotropy compared with single-network hydrogels [38,39,40,41,42,43,44,45]. Using our previously established silk fiber-reinforced IPN platform as a model system [38], the present study investigates whether vascular structural motifs can serve as independent design parameters for programming pressure-dependent vascular mechanics.

**Figure 1:**
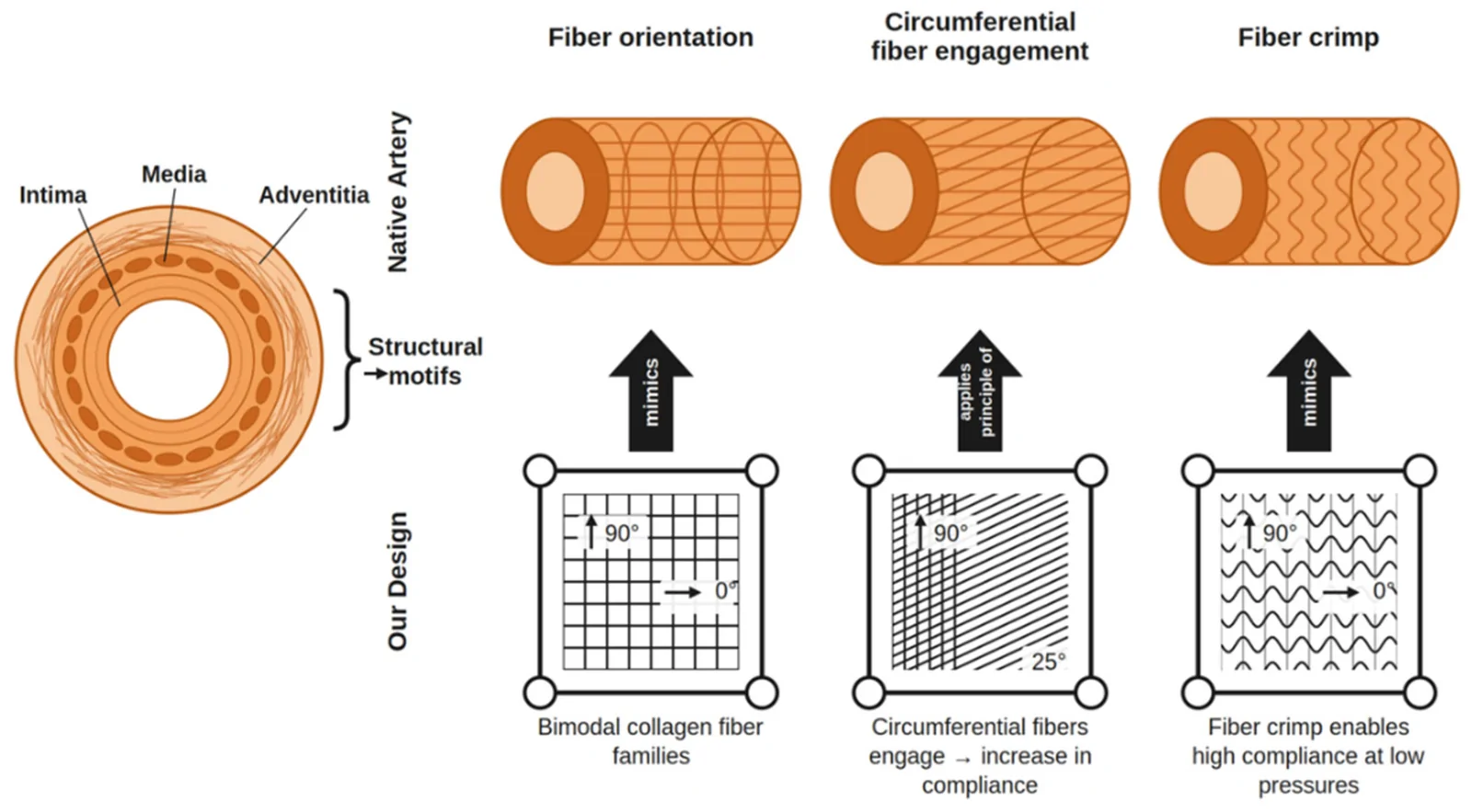
Bio-inspired fiber architecture design. The coronary artery wall (left) consists of three layers—intima, media, and adventitia—with the adventitia exhibiting key structural motifs that govern arterial mechanics: bimodal fiber orientation, circumferential fiber engagement under pressure, and collagen fiber crimp. Each motif was translated into a corresponding synthetic silk fiber architecture, fabricated by wrapping fibers around a rectangular frame: cross-plied (CP), reoriented (25°), and crimped CP, respectively. Upward arrows indicate that each synthetic design mimics (or, for circumferential engagement, applies the underlying mechanical principle of) its corresponding native structural motif.

Specifically, three architectures were examined: a baseline cross-plied (CP) configuration, a 25° fiber-reoriented configuration, and a crimped CP configuration (Figure 1). The pressure–diameter response of each construct was evaluated under internal pressurization and compared with reported data for young and aged coronary arteries [12,13,36,46]. We hypothesized that fiber reorientation and crimp-induced delayed fiber recruitment would increase compliance relative to the baseline CP architecture, demonstrating that structural motifs can systematically direct vascular mechanical behavior toward distinct physiological targets. By establishing structural motifs as programmable design parameters, this work contributes to biomimetics, a generalizable strategy for transferring the recruitment-based mechanical principles of native arterial tissue into synthetic fiber-reinforced constructs.

## 2. Materials and Methods

### 2.1. IPN Hydrogel Preparation

Alginate–polyacrylamide (Alg-PAAm) interpenetrating polymer network (IPN) hydrogel was prepared using a two-stage crosslinking process, consisting of covalent polyacrylamide polymerization followed by ionic alginate crosslinking. Briefly, a pre-gel solution was prepared by dissolving sodium alginate (2 wt%, Protanal^®^ LF 10-60, FMC Biopolymer, Philadelphia, PA, USA) and acrylamide (12 wt%, Sigma-Aldrich, Jerusalem, Israel) in double-distilled water (DDW). For each 20 mL of pre-gel solution, 1 mL of N,N′-methylenebisacrylamide (MBAA, 0.23 wt%, Sigma-Aldrich) was added as the covalent crosslinker, followed by 220 μL of ammonium persulfate (APS, 0.1 M, Sigma-Aldrich) as the thermal initiator and 40 μL of N,N,N′,N′-tetramethylethylenediamine (TEMED, Sigma-Aldrich) as the polymerization accelerator.

The solution was used immediately after the addition of APS and TEMED to prevent premature polymerization. Samples were first placed in an oven at 50 °C for 1 h to complete thermally induced PAAm polymerization. Following polymerization, the samples were immersed in 0.1 M CaCl_2_ solution for at least 48 h at room temperature to ionically crosslink the alginate network. All samples were maintained in 0.1 M CaCl_2_ solution until testing to preserve hydration and maintain the ionically crosslinked alginate network. The 2 wt% alginate/12 wt% acrylamide composition was adopted from the well-established tough alginate–polyacrylamide IPN system first reported by Sun et al. [40], in which this composition was shown to produce a highly stretchable and tough double-network hydrogel, and which we subsequently carried into our silk fiber-reinforced IPN platform [38]. As the aim of the present study was to isolate the effect of fiber architecture rather than matrix composition, the same matrix formulation was held constant across all constructs; composition itself was therefore not a variable optimized in this work.

### 2.2. Silk Fiber-Reinforced Laminate Fabrication

Silk fiber-reinforced hydrogel laminates were fabricated by embedding continuous Bombyx mori silk fibers within the Alg-PAAm IPN hydrogel matrix. Silk cocoons were partially degummed in double-distilled water (DDW) at 100 °C for 30 min, rinsed three times with DDW at room temperature, and stored in fresh DDW until use. Prior to laminate fabrication, silk fibers were gently pulled manually from the hydrated cocoons and wrapped around a custom rectangular frame to achieve the desired architecture.

After fiber placement, the frame containing the aligned silk fibers was positioned between two glass slides separated by spacers to control laminate thickness. Freshly prepared Alg-PAAm pre-gel solution was then poured between the glass slides until the fibers were fully submerged. The glass slides were held tightly together to maintain a uniform laminate geometry during polymerization. The fiber-containing hydrogel precursor was placed in an oven at 50 °C for 1 h to complete thermally induced PAAm polymerization, then immersed in a 0.1 M CaCl_2_ solution at room temperature for at least 48 h to induce alginate crosslinking. Following complete crosslinking, the silk fiber-reinforced laminates were gently separated from the glass slides and stored in 0.1 M CaCl_2_ solution until further processing.

Three fiber architectures were fabricated using the same general laminate preparation procedure, while varying the fiber-wrapping pattern or the post-wrapping fiber geometry. The cross-plied (CP) architecture was used as the baseline configuration. In this architecture, silk fibers were wrapped around the rectangular frame in two orthogonal directions, corresponding to 0° and 90° fiber families. This configuration was selected to provide a simplified biaxial reinforcement pattern and served as the reference architecture for evaluating the effects of fiber reorientation and crimping.

To investigate the effect of fiber orientation on pressure-dependent deformation, a reoriented architecture was fabricated by modifying the wrapping angle of one fiber family. In this configuration, one fiber family was aligned with the longitudinal axis of the future tubular construct, while the second fiber family was oriented at 25° relative to the circumferential inflation direction. The 25° architecture was designed to delay direct circumferential fiber engagement during inflation, allowing fibers to progressively rotate toward the hoop direction as the construct expanded. Fiber orientation angles in the fabricated laminates were measured from optical images using ImageJ (version 1.54g; National Institutes of Health, Bethesda, MD, USA).

To investigate the effect of fiber crimp on delayed fiber recruitment, a crimped CP architecture was fabricated. For this configuration, a CP fiber network was first prepared as described above and then placed within a custom two-part 3D-printed mold designed to impose a crimped fiber geometry. The mold was secured using an external clip and placed in an oven at 80 °C for 30 min to thermally fix the crimped configuration prior to hydrogel embedding. The mold cavity defined a sinusoidal crimp waveform with a nominal wavelength of 3.0 mm and a peak-to-peak amplitude of 2.0 mm, as specified by the mold’s CAD geometry. Following thermal fixation, the crimped fiber network was embedded within the Alg-PAAm IPN hydrogel matrix and crosslinked using the same polymerization and ionic crosslinking procedure described above. The crimp geometry of the fabricated laminates was verified by imaging the crimped constructs in both the bare (fiber-only) and hydrogel-embedded, hydrated states. The bare crimped fibers were imaged by macro-photography, and the hydrogel-embedded construct was imaged using the same digital camera setup used for the other architectures. Crimp wavelength was quantified from intensity line profiles taken across the fibers in ImageJ, with the image scale set from an in-plane reference; the spacing between successive intensity peaks corresponds to the crimp period.

Notably, all silk fibers across the three architectures were first degummed at 100 °C for 30 min prior to laminate fabrication; the 80 °C crimp-fixation step therefore did not expose the fibers to a higher temperature than their common processing history. Moreover, this temperature is well below the glass transition (~175–205 °C) and thermal degradation onset (~250 °C) of *Bombyx mori* silk fibroin [47,48], such that the load-bearing β-sheet crystalline structure of the fibers is not altered; at 80 °C the only expected thermal effect is the reversible removal of bound water, which is restored upon rehydration in CaCl_2_ prior to testing.

**Figure 2:**
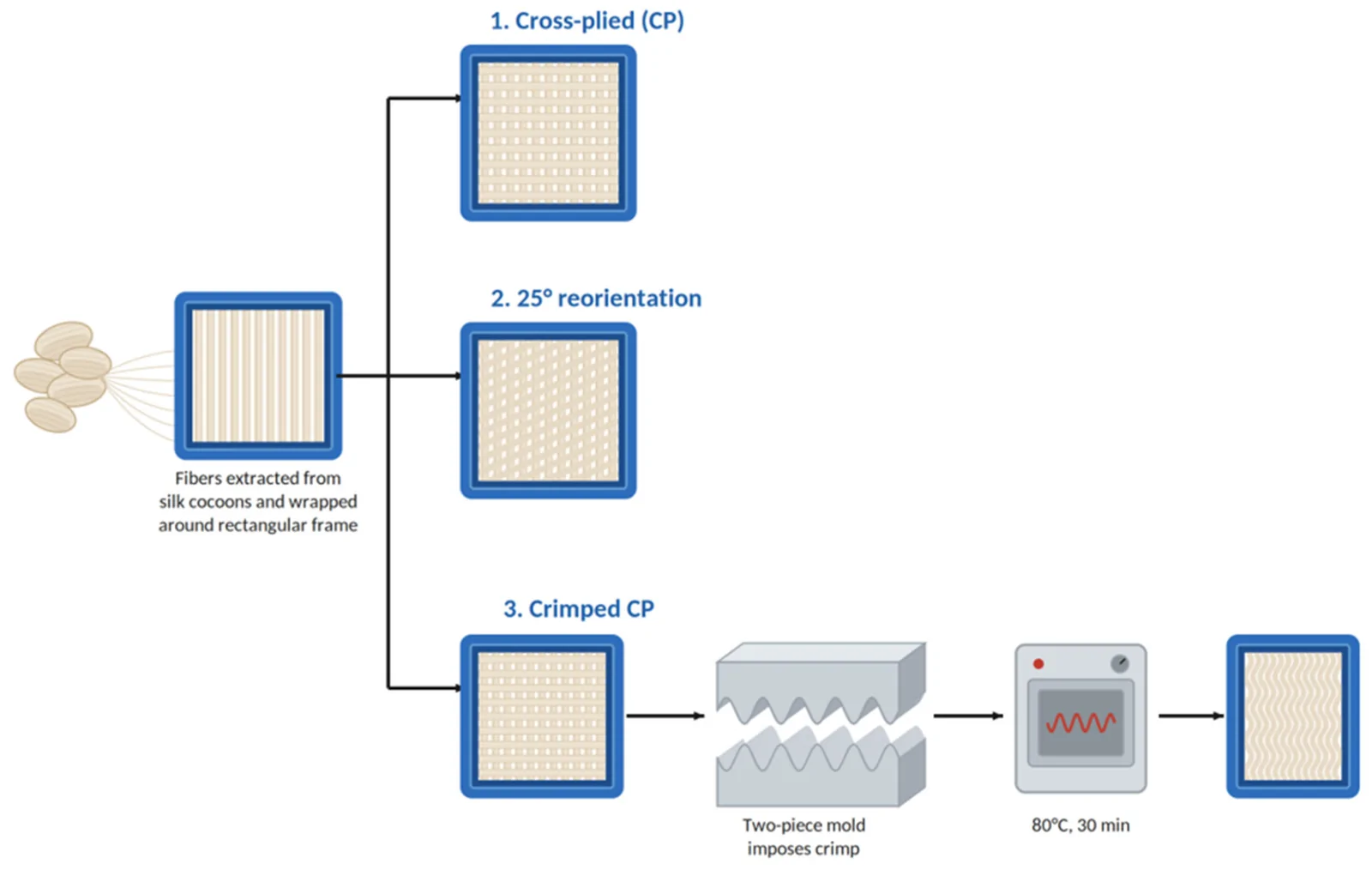
Fabrication of architecture-controlled silk fiber-reinforced hydrogel laminates. Silk fibers were extracted from Bombyx mori cocoons and wrapped around rectangular frames to generate predefined reinforcement architectures. The wrapped fibers were then divided into three configurations: cross-plied (CP), consisting of orthogonal 0°/90° fiber families; 25° reorientation, in which an angled fiber family was wrapped over the 0° fibers at 25° relative to the circumferential direction; and crimped CP, in which a CP fiber frame was placed in a custom two-piece mold to impose a wavy fiber geometry, followed by thermal fixation at 80 °C for 30 min. These architectures were subsequently embedded within the Alg-PAAm IPN hydrogel matrix to investigate the effects of fiber orientation and crimp on tensile and pressure-dependent mechanical behavior.

Thus, all investigated architectures were composed of the same silk fiber-reinforced Alg-PAAm IPN material system, with the organization of the reinforcing fibers serving as the primary design variable distinguishing them: orthogonal CP fibers, 25° reoriented fibers, or crimped CP fibers (Figure 2). Although the crimped configuration additionally required a mild thermal fixation step, fiber organization is expected to be the dominant factor underlying the observed mechanical differences (see Section 2.2). This enabled the effect of structural motifs on tensile and pressure-dependent mechanical behavior to be isolated from changes in material composition.

### 2.3. Tubular Sample Fabrication

Following fabrication and crosslinking of the planar laminates, a portion of the samples from each investigated architecture was prepared for tensile testing, while the remaining laminates were converted into tubular constructs for pressure-dependent mechanical evaluation. Tubular samples were fabricated by manually wrapping the laminates around a cylindrical rod with a diameter of 4 mm. The overlapping region of the laminate was subsequently subjected to an additional crosslinking step to stabilize the tubular geometry prior to testing (Table 1).

### 2.4. Tensile Testing

Uniaxial tensile tests were performed on rectangular laminate samples from each architecture using a μTS tensile testing system (Psylotech, Evanston, IL, USA) equipped with a 222 N load cell. All samples were maintained in 0.1 M CaCl_2_ solution until testing to preserve hydration. Prior to testing, sample width and thickness were measured using a digital caliper and micrometer, respectively, and were used to calculate the initial cross-sectional area (Table 2).

Samples were mounted between custom 3D-printed clamps, and the initial gauge length was defined as the distance between the clamp edges. A preload of 1 N was applied prior to testing. Tensile tests were conducted at a constant strain rate of 1% s^−1^. Following preload, samples were subjected to three preconditioning cycles to 5% strain and were then stretched to failure.

Engineering stress was calculated as the applied force divided by the initial cross-sectional area, and engineering strain was calculated as the change in length divided by the initial gauge length. The elastic modulus was extracted from the linear region of the stress–strain curve over a strain window selected individually for each architecture to fall beyond its respective toe-to-linear transition (identified as described in the toe-region analysis below). Ultimate tensile strength (UTS) was defined as the peak stress, and maximum strain was defined as the corresponding strain at UTS. Toughness was calculated as the area under the stress–strain curve.

### 2.5. Toe-Region Analysis

To quantify the low-stiffness toe region preceding fiber engagement, each tensile stress–strain curve was truncated at the strain corresponding to UTS, resampled onto a uniform 200-point strain grid, and lightly smoothed (moving average, window ≈6% of curve length) to remove instrument noise. The strain at which the tangent stiffness (the local slope, *dσ*/*dε*, of the smoothed curve) reached its maximum value, *ε_peak_*, was identified for each replicate.

A bilinear (two-segment) least-squares regression was then fit to the curve over the range from zero strain to $\varepsilon_{peak}$, by minimizing the combined residual sum of squares of two linear segments meeting at a candidate breakpoint strain $\varepsilon_{b}$:

(1) $$\sigma \left(\varepsilon \right)=\{\begin{array}{c} E_{toe}\varepsilon +c_{1},\;\;\;\;\;\;\varepsilon \leq \varepsilon_{b}\\ E_{lin}\varepsilon +c_{2},\;\;\;\;\;\;\;\varepsilon >\varepsilon_{b} \end{array}$$

where *E_toe_* and *E_lin_* are the elastic moduli of the toe and linear segments, *c*_1_ and *c*_2_ are their respective intercepts, and the summation runs over all data points in the fitted range. Each candidate breakpoint $\varepsilon_{b}$ within the search range (15–90% of the points preceding *ε_peak_*) was tested, and the breakpoint giving the best-fitting bilinear description of the curve was taken as the strain marking the end of the toe region.

The relative sharpness of the toe-to-linear transition, independent of absolute stiffness, was expressed as:

(2) $$\frac{E_{lin}}{E_{toe}}$$

Toe energy was calculated as the area under the stress–strain curve from zero strain to the toe strain:

(3) $$U_{toe}=\int_{0}^{\varepsilon_{b}}\sigma (\varepsilon )d\varepsilon$$

evaluated numerically by trapezoidal integration of the smoothed curve.

### 2.6. Pressure–Diameter Testing

**Figure 3:**
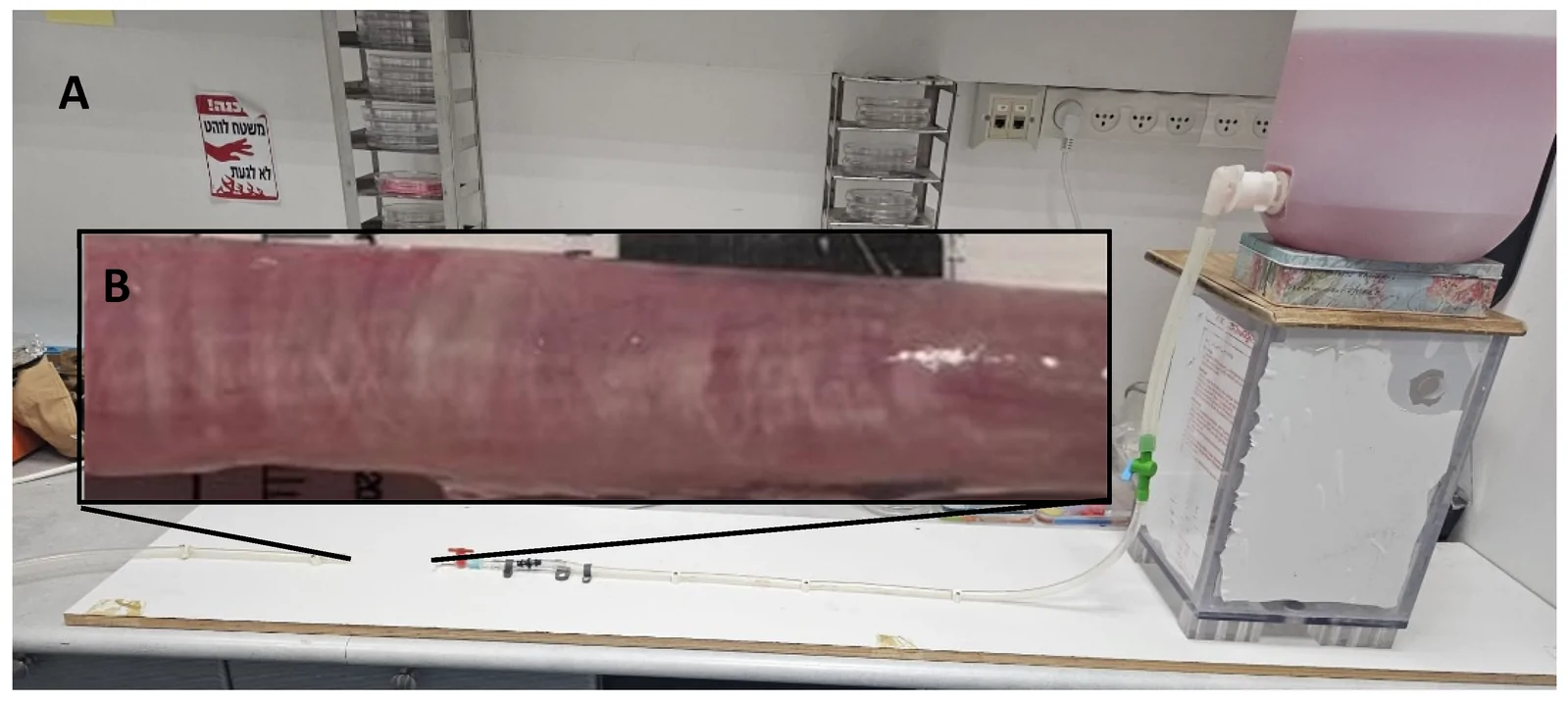
(**A**) Experimental setup for internal pressure testing of the tubular samples. (**B**) A representative tubular sample.

Pressure-dependent deformation behavior was evaluated using a custom-built static inflation setup (Figure 3) designed to measure changes in the external diameter of tubular constructs under controlled internal pressure. The setup consisted of a vertically adjustable liquid reservoir connected to the tubular sample through flexible tubing. Internal pressure was generated hydrostatically by changing the height of the liquid column relative to the sample, according to:

(4) P=ρgh

where *P* is the applied pressure, *ρ* is the density of the liquid, *g* is the gravitational acceleration, and *h* is the height difference between the liquid level in the reservoir and the sample. The reservoir height was adjusted to apply predefined pressure levels, allowing controlled inflation of the tubular constructs under quasi-static loading conditions.

Each tubular construct was mounted horizontally and connected at both ends to the pressure system using custom connectors. Care was taken to avoid twisting or axial stretching of the tube during mounting. Prior to testing, the system was filled and inspected for leakage. The sample was then gradually pressurized from 0 to 160 mmHg in 10 mmHg increments. At each pressure step, the pressure was allowed to stabilize before image acquisition.

The external diameter of the tubular construct was recorded at each pressure level using a digital microscope camera (AM-01, ANNLOV; 4.3-inch LCD, 50–1000× digital magnification). A calibration film with known dimensions was positioned beneath the sample and imaged together with the construct to provide a pixel-to-length conversion for subsequent image analysis. Image calibration using the reference film yielded a spatial resolution of 19.2 µm/pixel (52 pixels/mm).

For each pressure step, images were analyzed in ImageJ (NIH, Bethesda, MD, USA) to extract the external diameter of the construct. Diameter measurements were taken from the central region of the tube to minimize edge effects associated with the connectors. The measured diameter at each pressure was normalized to the diameter measured at 50 mmHg, which was defined as the lower bound of the physiological pressure range:

(5) $$D/D_{50}$$

where *D* is the measured external diameter at a given pressure and *D*_50_ is the external diameter measured at 50 mmHg. This normalization enabled direct comparison between samples with small differences in initial diameter and allowed comparison with reported pressure–diameter behavior of native coronary arteries. A minimum of four independent tubular constructs were tested for each architecture (*n* = 4).

### 2.7. Curve-Shape Similarity to Native Arteries

To complement the single-pressure-point comparison at 160 mmHg, the similarity between each construct’s pressure–diameter curve and the native artery reference curves, including their reported variability (mean ± SD, digitized from [12,13]), was quantified across the entire measured pressure range. For each pressure point, a normalized distance between the construct and native curves was calculated as the difference between their means divided by their combined SD; a value ≤ 1 indicates the two curves are statistically indistinguishable at that pressure. Averaging this normalized distance across the measured range gave a Native Similarity Score (%) for each replicate against each native reference, where 100% indicates the construct curve sits at the center of the native distribution and 0% indicates it sits, on average, at the edge of statistical overlap. The Native Similarity Score is intended as a descriptive index of curve proximity across the full pressure range, not as an inferential statistical test; it summarizes how closely each construct tracks the native reference distribution rather than establishing statistical equivalence.

### 2.8. Compliance Calculation

Compliance values were calculated from the pressure-dependent external diameter measurements obtained during inflation testing. Circumferential compliance was determined using the following equation:

(6) $$C=\frac{(D_{2}-D_{1})/D_{1}}{P_{2}-P_{1}}\times 10^{4}$$

where $D_{1}$ and $D_{2}$ represent the external diameters measured at pressures $P_{1}$ and $P_{2}$, respectively. Compliance values were reported as %/100 mmHg.

Compliance was calculated for each 10 mmHg pressure interval throughout the investigated pressure ranges. In addition, average compliance values were determined for the physiological pressure ranges of 50–90, 80–120, and 110–150 mmHg, corresponding to hypotensive, normotensive, and hypertensive blood pressures, respectively.

### 2.9. Optical Imaging

Optical microscope images of the laminates were acquired using an industrial camera (uEye IDS U3-3280CP-C-HQ rev. 2.2, IDS Imaging Development Systems GmbH, Obersulm, Germany) with an Electric linear zoom lens X12.0 (Mvotem Optics, Kunshan, China).

### 2.10. Statistical Analysis

Statistical significance between architectures was determined using one-way analysis of variance (ANOVA) with Tukey’s post hoc test (*p* < 0.05) for tensile mechanical parameters (modulus, UTS, maximum strain, toughness) and for compliance at each pressure range. One-sample *t*-tests were used to compare each architecture’s *D/D*_50_ value at 160 mmHg against reference values from the literature for young and aged human coronary arteries. All statistical analyses were performed using GraphPad Prism 10^®^.

## 3. Results

### 3.1. Biomimetic Structural Motifs for Tuning Vascular Mechanics

**Figure 4:**
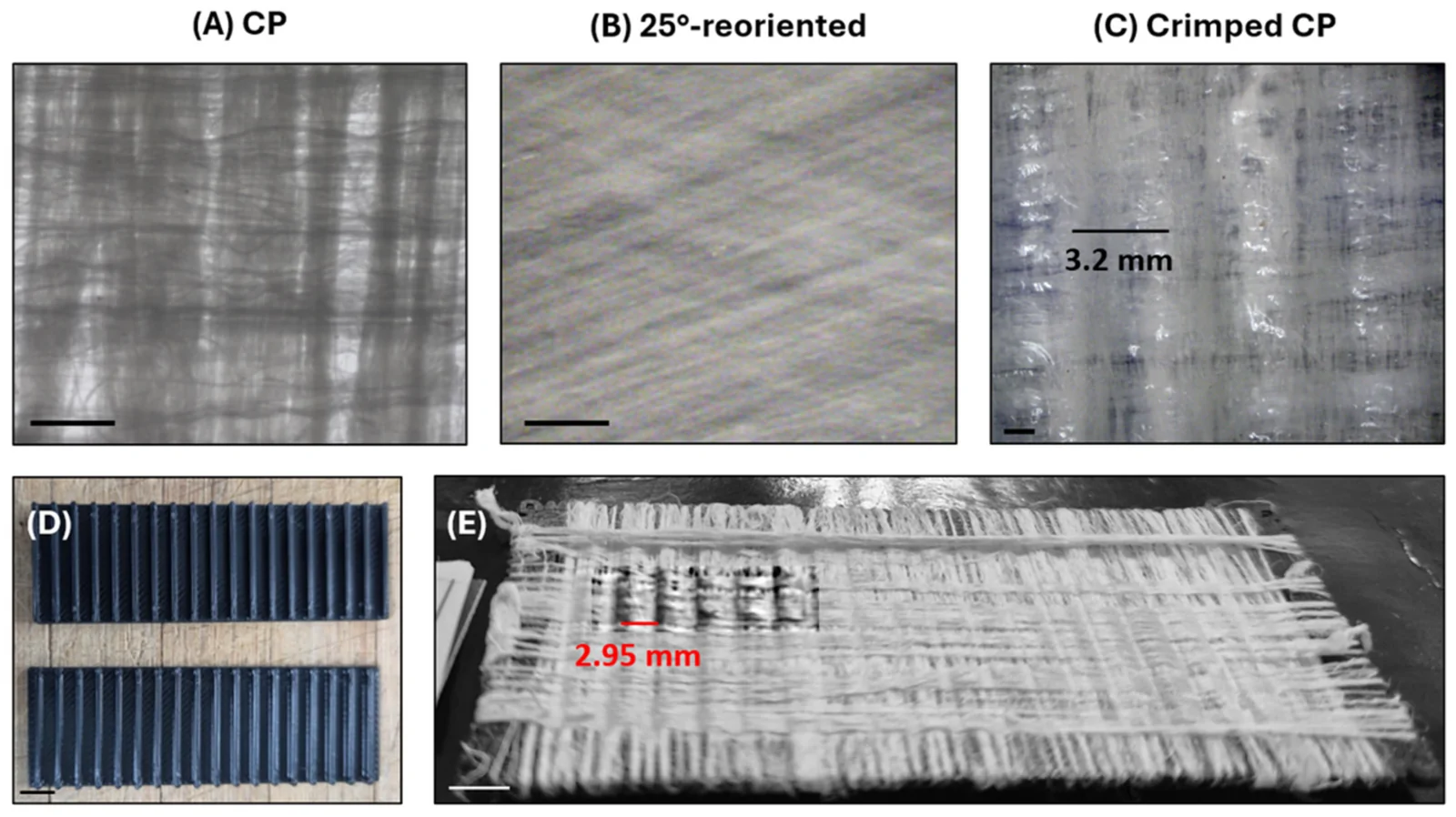
Fabricated silk fiber-reinforced laminate architectures and the crimping mold. (**A**) Cross-plied (CP) architecture, showing the orthogonal fiber families. (**B**) 25–reoriented, in which the fibers are offset to 25° relative to the circumferential direction. The measured angle between the two fiber families was approximately 25.3° (measured from optical images using ImageJ). (**C**) Crimped CP architecture embedded within the hydrated hydrogel, with a representative crimp-wavelength marker. (**D**) Two-piece mold used to impose the sinusoidal crimp on the CP laminate; the mold teeth have a 3.0 mm period and a 2.0 mm peak-to-peak height, defining the nominal crimp geometry. (**E**) Bare crimped fibers, with a representative crimp-wavelength marker. Crimp wavelength was quantified from intensity line profiles taken across the fibers in ImageJ; the marked segments in (**C**,**E**) are representative and drawn to scale. The measured crimp wavelength was 2.95 ± 0.28 mm for the bare fibers (**E**) and 3.20 ± 0.12 mm for the embedded, hydrated construct (**C**), both in close agreement with the nominal mold-defined wavelength of 3.0 mm, confirming that the crimp wavelength is preserved through thermal fixation, hydrogel embedding, and hydration. Scale bars: (**A**–**C**) 1 mm; (**D**,**E**) 5 mm.

Three tubular fiber architectures were designed to investigate how structural motifs influence the mechanical response of the constructs. A baseline cross-plied (CP) architecture was used as the reference configuration. Two additional architectures were introduced through structural modification of the fiber arrangement: a reoriented architecture with fibers aligned at 25° relative to the circumferential direction and a CP architecture with manually induced fiber crimp of nominal wavelength 3.0 mm and peak-to-peak amplitude 2.0 mm. To confirm that the intended crimp geometry was achieved in the fabricated constructs, the crimp wavelength was quantified from intensity line profiles taken across the fibers in ImageJ (Figure 4). The bare crimped fibers were imaged by macro-photography and the hydrogel-embedded, hydrated construct was imaged using the same setup as the other architectures in Figure 4. The measured wavelength was 2.95 ± 0.28 mm for the bare crimped fibers and 3.20 ± 0.12 mm for the fibers embedded in the hydrated hydrogel, both in close agreement with the nominal mold-defined wavelength of 3.0 mm. This confirms that the crimp wavelength is preserved through thermal fixation, hydrogel embedding, and hydration.

The selected architectures were designed to mimic structural features associated with native arterial tissue, including fiber orientation and crimp-mediated recruitment behavior. The effect of these structural modifications on the tensile and pressure-dependent mechanical response of the constructs was subsequently evaluated. Representative optical images of the fabricated laminates for each architecture are shown in Figure 4.

### 3.2. Effect of Fiber Architecture on Tensile Mechanical Behavior

**Figure 5:**
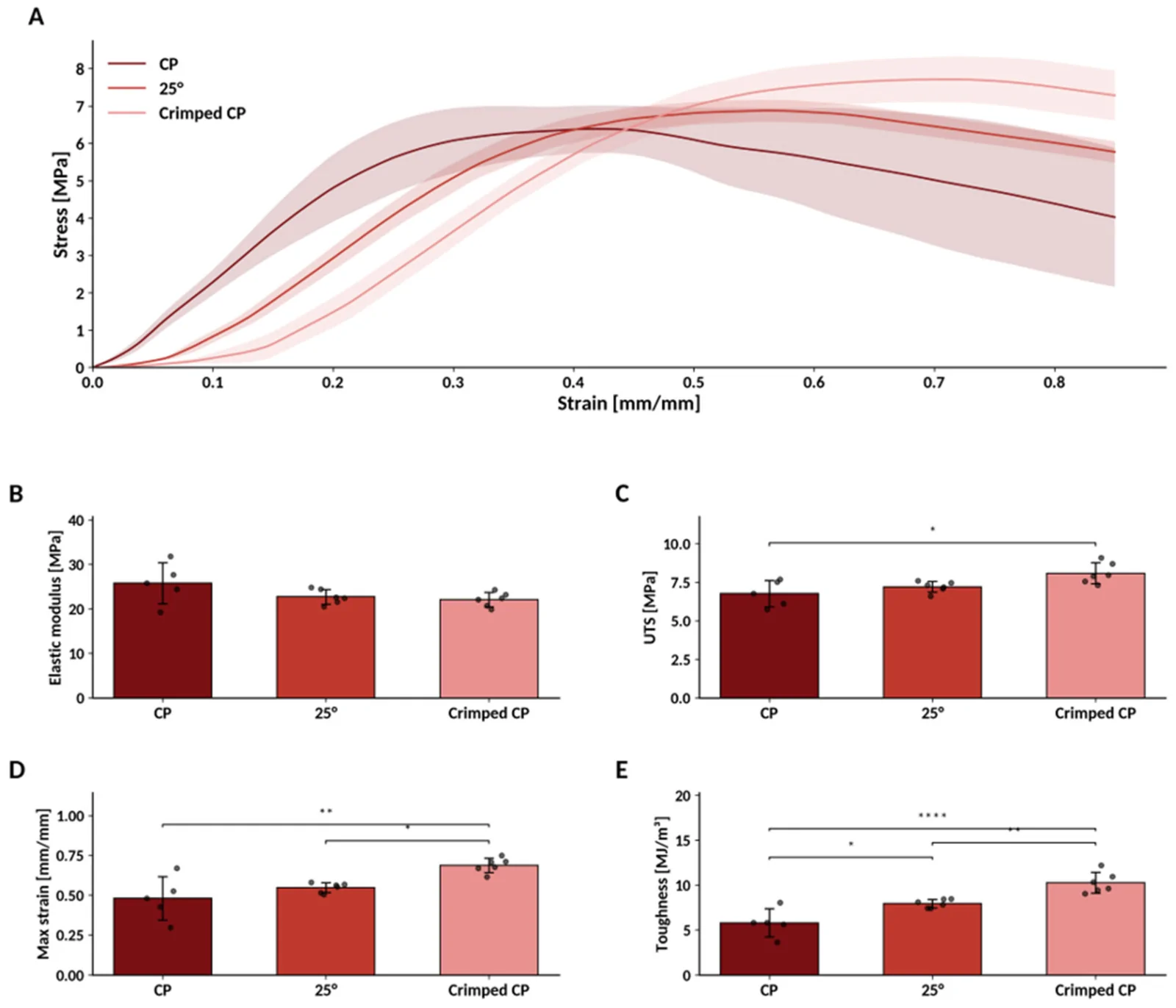
Architecture-dependent tensile mechanical behavior. (**A**) Uniaxial stress–strain curves for CP, 25°, and Crimped CP architectures (mean ± SD, shown in red shades). Tensile parameters: elastic modulus (**B**), ultimate tensile strength (**C**), maximum strain (**D**), and toughness (**E**), shown as mean ± SD with individual data points overlaid. Asterisks indicate statistically significant differences between architectures (* *p* < 0.05, ** *p* < 0.01, **** *p* < 0.0001).

The tensile mechanical response varied between the investigated architectures (Figure 5). Elastic modulus did not differ significantly between architectures (CP: 25.78 ± 4.59 MPa; 25°: 22.72 ± 1.64 MPa; Crimped CP: 22.10 ± 1.61 MPa, *p* = 0.108), indicating that structural modification of the fiber architecture did not come at the cost of construct stiffness. In contrast, UTS increased significantly with crimping, from 6.77 ± 0.85 MPa (CP) to 8.09 ± 0.68 MPa (Crimped CP) (*p* = 0.012), while the 25° architecture was intermediate (7.21 ± 0.35 MPa) and not significantly different from either configuration. Maximum strain increased progressively across the architectures, from 0.48 ± 0.14 (CP) to 0.55 ± 0.03 (25°) to 0.69 ± 0.05 (Crimped CP), with the Crimped CP architecture significantly exceeding both CP (*p* = 0.002) and 25° (*p* = 0.022). Toughness exhibited the clearest architecture-dependent trend, increasing monotonically from 5.78 ± 1.56 MJ/m^3^ (CP) to 7.93 ± 0.47 MJ/m^3^ (25°) to 10.26 ± 1.17 MJ/m^3^ (Crimped CP). These results demonstrate that structural modification of fiber architecture enhances strain capacity and energy dissipation without compromising construct stiffness and that crimp specifically increases tensile strength relative to the CP baseline.

### 3.3. Structural Architecture Tunes the Toe Region of the Tensile Response

**Figure 6:**
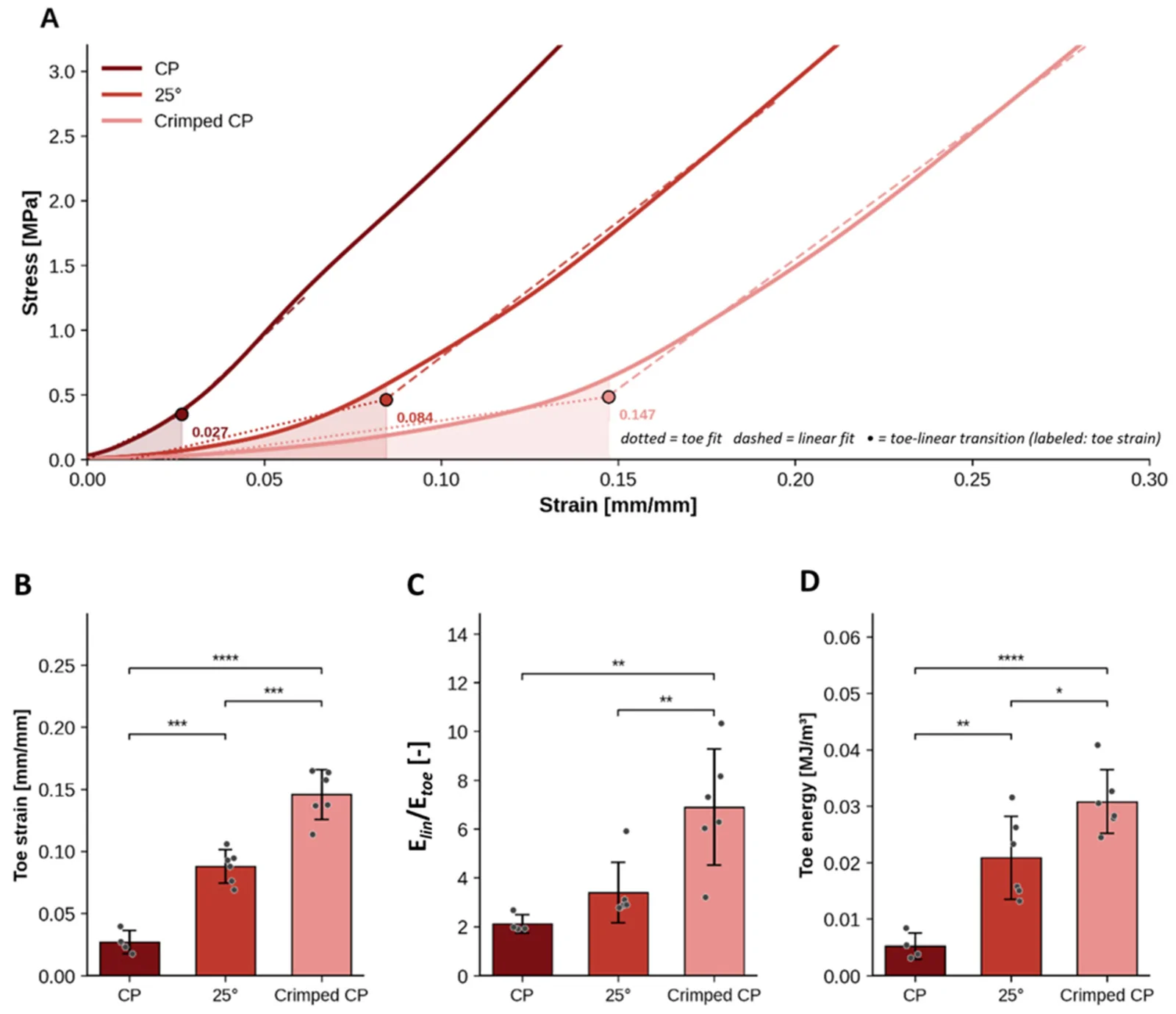
Quantification of the toe region preceding fiber engagement. (**A**) Representative mean stress–strain curves (CP, 25°, Crimped CP; red shades) zoomed to the toe region. Dotted lines show the fitted toe-region modulus (*E_toe_*); dashed lines show the fitted linear-region modulus (*E_lin_*); filled circles mark the toe-to-linear transition (toe strain, labeled); shaded areas indicate toe energy. Toe strain (**B**), *E_lin_*/*E_toe_* ratio (**C**), and toe energy (**D**), shown as mean ± SD with individual data points overlaid (CP: *n* = 6; 25°: *n* = 6; Crimped CP: *n* = 6). Asterisks indicate statistically significant differences between architectures (* *p* < 0.05, ** *p* < 0.01, *** *p* < 0.001, **** *p* < 0.0001).

To quantify the toe region visible in the stress–strain curves (Figure 6A), the low-stiffness toe and higher-stiffness linear regions were resolved for each replicate and architecture. Toe strain increased progressively and significantly across all three architectures, from 0.027 ± 0.009 mm/mm (CP) to 0.088 ± 0.013 mm/mm (25°) to 0.146 ± 0.020 mm/mm (Crimped CP) (*p* < 0.0001). The *E_lin_*/*E_toe_* ratio, which expresses the sharpness of the toe-to-linear transition independent of absolute stiffness, likewise increased from 2.12 ± 0.38 (CP) to 3.39 ± 1.24 (25°) to 6.89 ± 2.38 (Crimped CP) (*p* = 0.00135); the increase was statistically significant for Crimped CP relative to both CP (*p* = 0.0019) and 25° (*p* = 0.0081), but the difference between CP and 25° alone did not reach significance (*p* = 0.486). Toe energy followed the same monotonic and significant trend, increasing from 0.0052 ± 0.0023 MJ/m^3^ (CP) to 0.0208 ± 0.0073 MJ/m^3^ (25°) to 0.0308 ± 0.0057 MJ/m^3^ (Crimped CP) (*p* < 0.0001). Taken together, these results indicate that fiber reorientation and, more markedly, crimp induction extend the low-stiffness toe region to progressively higher strains and sharpen the subsequent transition into the stiffer linear region, consistent with a structurally delayed onset of fiber load-bearing.

### 3.4. Pressure–Diameter Response Under Physiological Loading

**Figure 7:**
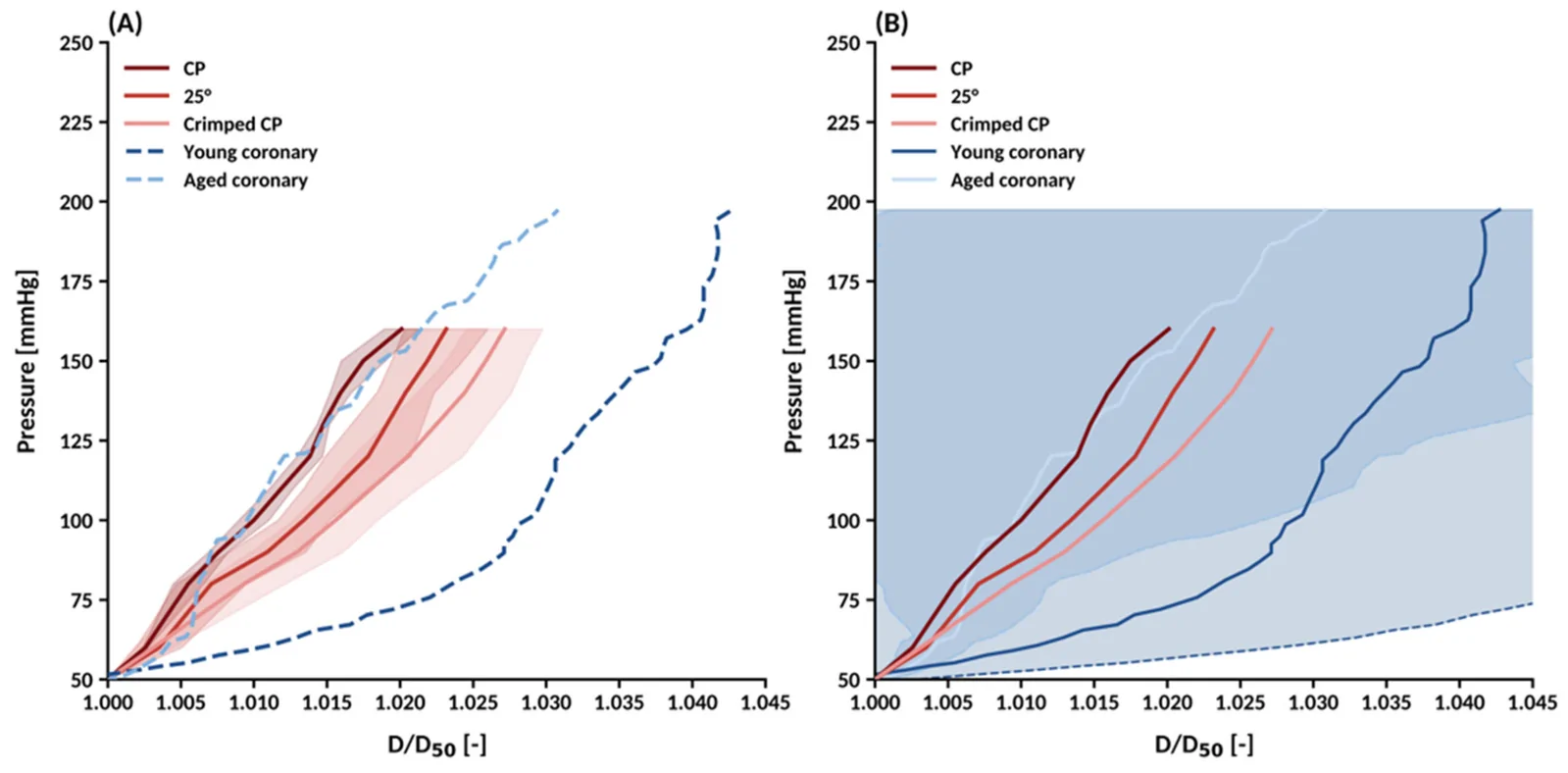
Pressure–diameter response of the investigated fiber architectures. Normalized outer diameter (*D/D*_50_) plotted against internal pressure for CP, 25°, and Crimped CP constructs (mean ± SD, *n* = 4 per architecture, shown in red shades), compared with reference curves from the literature for young and aged human coronary arteries [12,13,36]. Two complementary representations are shown. (**A**) Construct means with their across-replicate variability shown as shaded ± SD bands, overlaid on the native coronary mean curves (dashed lines). (**B**) Construct mean curves overlaid on the native coronary reference distributions, with each native reference shown as its mean (solid line) ± SD (shaded band with matching dashed outline). Construct compliance fell within native coronary artery variability across the entire measured pressure range for all three architectures. *D/D*_50_ is defined as 1.0 at 50 mmHg by construction; axes are therefore shown from this reference point. Native variability (mean ± SD) encompasses all construct responses across the measured pressure range.

The pressure–diameter response of the investigated architectures is presented in Figure 7. All constructs exhibited a progressive increase in normalized diameter (*D/D*_50_) as internal pressure increased, consistent with the nonlinear, pressure-dependent distension characteristic of arterial tissue. At 160 mmHg, the CP architecture reached a *D/D*_50_ value of 1.020 ± 0.001 (*n* = 4), closely tracking the reported behavior of aged coronary arteries (*D/D*_50_ = 1.0213 at the same pressure) across the tested pressure range. Fiber reorientation to 25° increased the normalized diameter response relative to CP, reaching a *D/D*_50_ value of 1.023 ± 0.003 (*n* = 4) at 160 mmHg. The Crimped CP architecture exhibited the greatest normalized diameter increase among the investigated constructs, reaching a *D/D*_50_ value of 1.027 ± 0.003 (*n* = 4) at 160 mmHg, positioning it between the aged and young coronary reference curves (*D/D*_50_ = 1.0397 for the young coronary at 160 mmHg) but distinct from both.

One-sample *t*-tests comparing each architecture’s *D/D_50_* value at 160 mmHg against the corresponding reference values from the literature indicated that the CP and 25° architectures did not differ significantly from aged coronary arteries (*p* = 0.223 and *p* = 0.281, respectively) but differed significantly from young coronary arteries (*p* = 0.0012 and *p* = 0.0014, respectively). The Crimped CP architecture differed significantly from both young (*p* = 0.0023) and aged (*p* = 0.020) coronary arteries, indicating that structural modification shifted the construct into a distinct intermediate mechanical regime rather than fully reaching either physiological target. These results demonstrate that fiber reorientation and crimp can be used to progressively tune the pressure-dependent response of the constructs along the continuum between aged and young arterial behavior.

### 3.5. Structural Architecture Tunes Curve-Shape Similarity to Native Arteries

**Figure 8:**
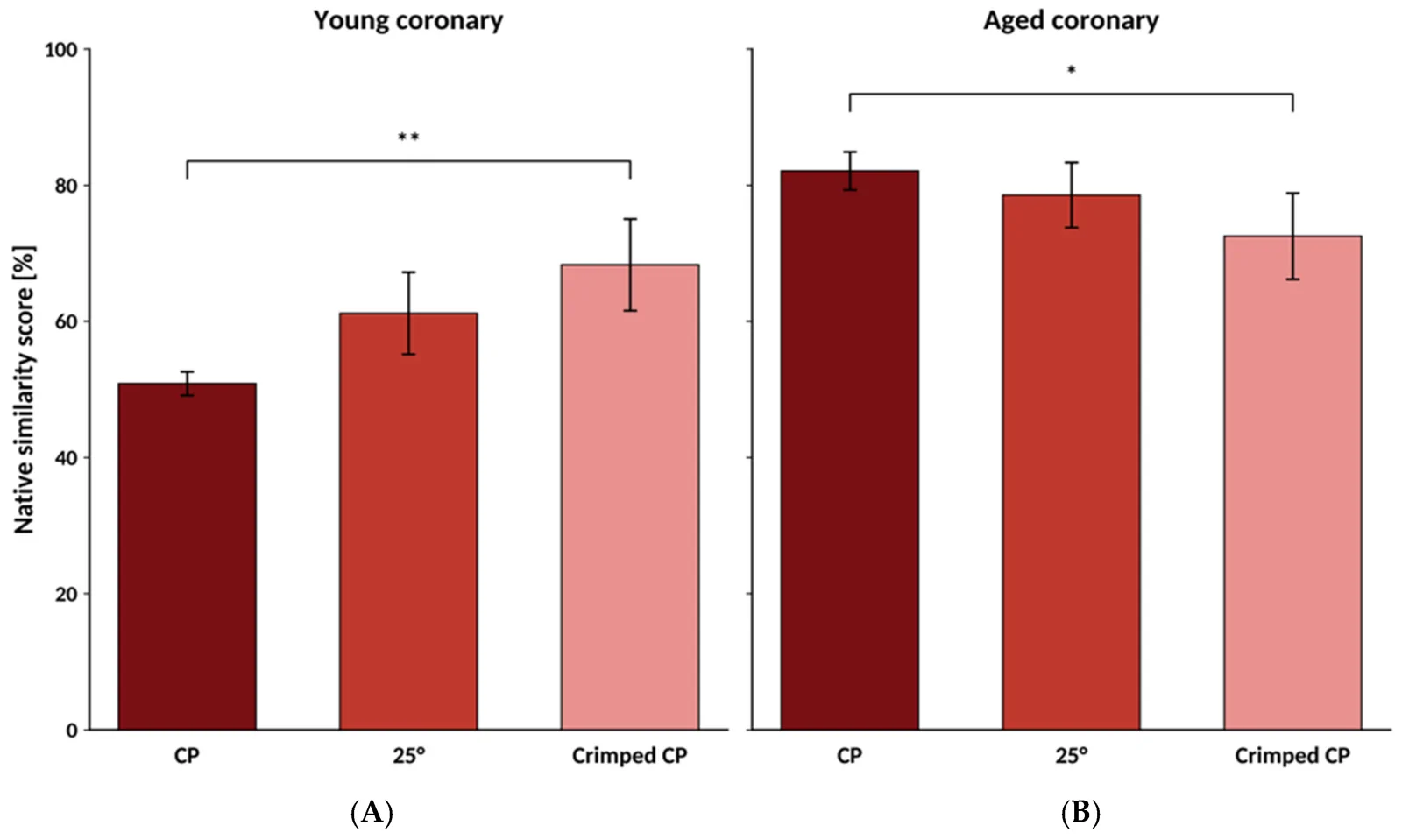
Native Similarity Score of the pressure–diameter response relative to native coronary arteries (see Methods), where 100% indicates the construct curve sits at the center of the native distribution and 0% indicates it sits, on average, at the edge of statistical overlap. (**A**) Similarity score relative to young coronary artery. (**B**) Similarity score relative to aged coronary artery. Data shown as mean ± SD (*n* = 4 per architecture). Asterisks indicate statistically significant differences between architectures (* *p* < 0.05, ** *p* < 0.01).

Because the one-sample *t*-tests above compare constructs to native arteries only at 160 mmHg, this comparison was extended across the full measured pressure range using the Native Similarity Score (see Methods), with results shown in Figure 8. Across all three architectures, construct compliance fell within the native coronary artery variability over the entire measured pressure range for both young and aged references. Similarity to the young coronary reference increased progressively from 50.9 ± 1.7% (CP) to 61.2 ± 6.0% (25°) to 68.3 ± 6.7% (Crimped CP). Conversely, similarity to the aged coronary reference decreased from 82.1 ± 2.8% (CP) to 78.6 ± 4.8% (25°) to 72.5 ± 6.3% (Crimped CP). These results confirm, across the full physiological pressure range, the same progressive trend observed at the single 160 mmHg endpoint.

### 3.6. Compliance Across Physiological Pressure Ranges

**Figure 9:**
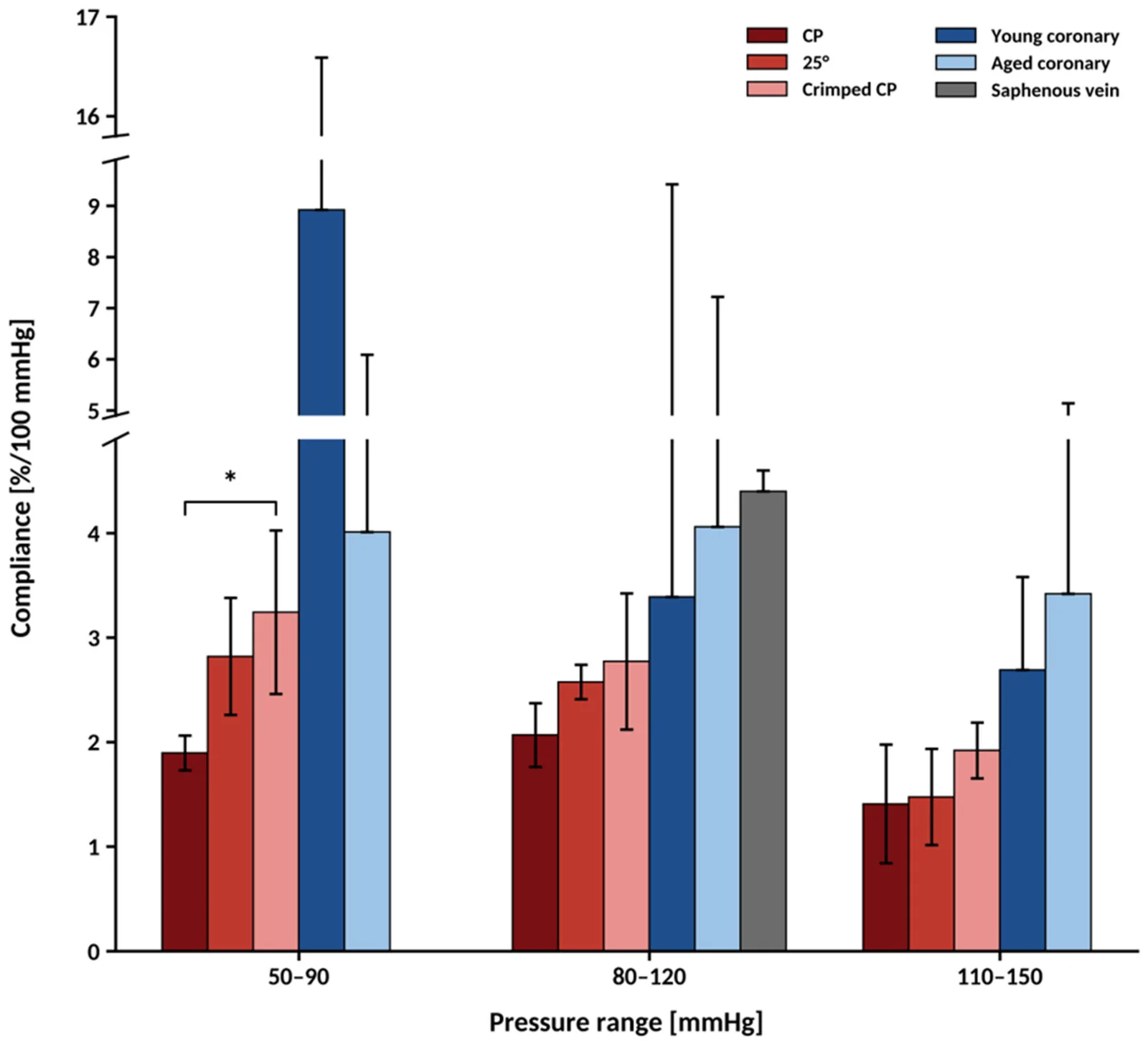
Compliance of the investigated fiber architectures across three physiological pressure ranges (hypotensive, 50–90 mmHg; normotensive, 80–120 mmHg; hypertensive, 110–150 mmHg), compared with literature reference values for young and aged human coronary arteries and the saphenous vein [12,36]. Bars represent mean ± SD (*n* = 4 per architecture; native tissue error bars shown one-sided due to large reported variance). The y-axis is broken (two discontinuities) to accommodate the substantially higher compliance and variability of the young coronary reference while preserving resolution among the construct groups; error bars extending across a break are shown truncated at the segment boundaries and continue in the segment above. (* *p* < 0.05); no significant differences were detected in the 80–120 and 110–150 mmHg ranges.

Compliance values for the investigated architectures across the three physiological pressure ranges are presented in Figure 9. In the hypotensive range (50–90 mmHg), compliance differed significantly between architectures (*p* = 0.0225), with the Crimped CP architecture (3.24 ± 0.78%/100 mmHg) exhibiting significantly higher compliance than the CP baseline (1.90 ± 0.17%/100 mmHg; *p* = 0.020). The 25° architecture (2.82 ± 0.56%/100 mmHg) was intermediate and not significantly different from either CP or Crimped CP.

In the normotensive (80–120 mmHg) and hypertensive (110–150 mmHg) ranges, the same directional trend was observed—compliance increased progressively from CP to 25° to Crimped CP—but the differences between architectures did not reach statistical significance (*p* = 0.107 and *p* = 0.271, respectively), likely reflecting the combined effects of smaller sample size and biological variability at these pressure ranges.

Comparison with native tissue compliance values reported in the literature showed that all three architectures remained below the young coronary artery (8.92 ± 7.67, 3.39 ± 6.03, and 2.69 ± 0.89%/100 mmHg for the 50–90, 80–120, and 110–150 mmHg ranges, respectively) and approached the aged coronary artery and saphenous vein ranges (4.01 ± 2.08 and 4.06 ± 3.16%/100 mmHg for aged coronary at 50–90 and 80–120 mmHg, respectively; 4.4 ± 0.2%/100 mmHg for saphenous vein at 80–120 mmHg). Notably, the values reported in the literature for native tissue compliance exhibited substantial variability, particularly at lower pressures, consistent with previously noted inconsistency in compliance measurements across studies. Taken together, these findings demonstrate that structural modification of the fiber architecture provides a significant and tunable mechanism for increasing compliance at low physiological pressures, with a consistent trend extending across the full physiological pressure range.

## 4. Discussion

The present study establishes an architecture-driven strategy for programming vascular mechanics through biomimetic structural motifs. Building on our previously developed silk fiber-reinforced IPN hydrogel platform [38], we demonstrate that fiber reorientation and crimp function as programmable engineering parameters that systematically tune pressure-dependent mechanical behavior without altering material composition. Fiber reorientation increased compliance relative to the baseline CP configuration, while crimp enhanced pressure-dependent diameter expansion, further supporting the growing evidence that vascular mechanics are governed not only by constituent materials but also by structural organization [18,21,29,36,38,49,50,51]. Together, these findings establish structural architecture as an independent engineering design axis that complements material composition in controlling tissue mechanics.

Fiber reorientation and crimp increased compliance while elastic modulus remained unchanged—most markedly at low physiological pressures, where crimped constructs showed significantly higher compliance than the CP baseline (3.24 vs. 1.90%/100 mmHg; *p* = 0.020)—demonstrating that these structural motifs enable systematic tuning of compliance without the conventional stiffness-compliance trade-off [52,53]. This is consistent with the broader concept that vascular graft mechanics can be programmed through architecture and that physiological relevance depends on the full deformation response rather than on modulus alone [9,49,50,52,53].

**Figure 10:**
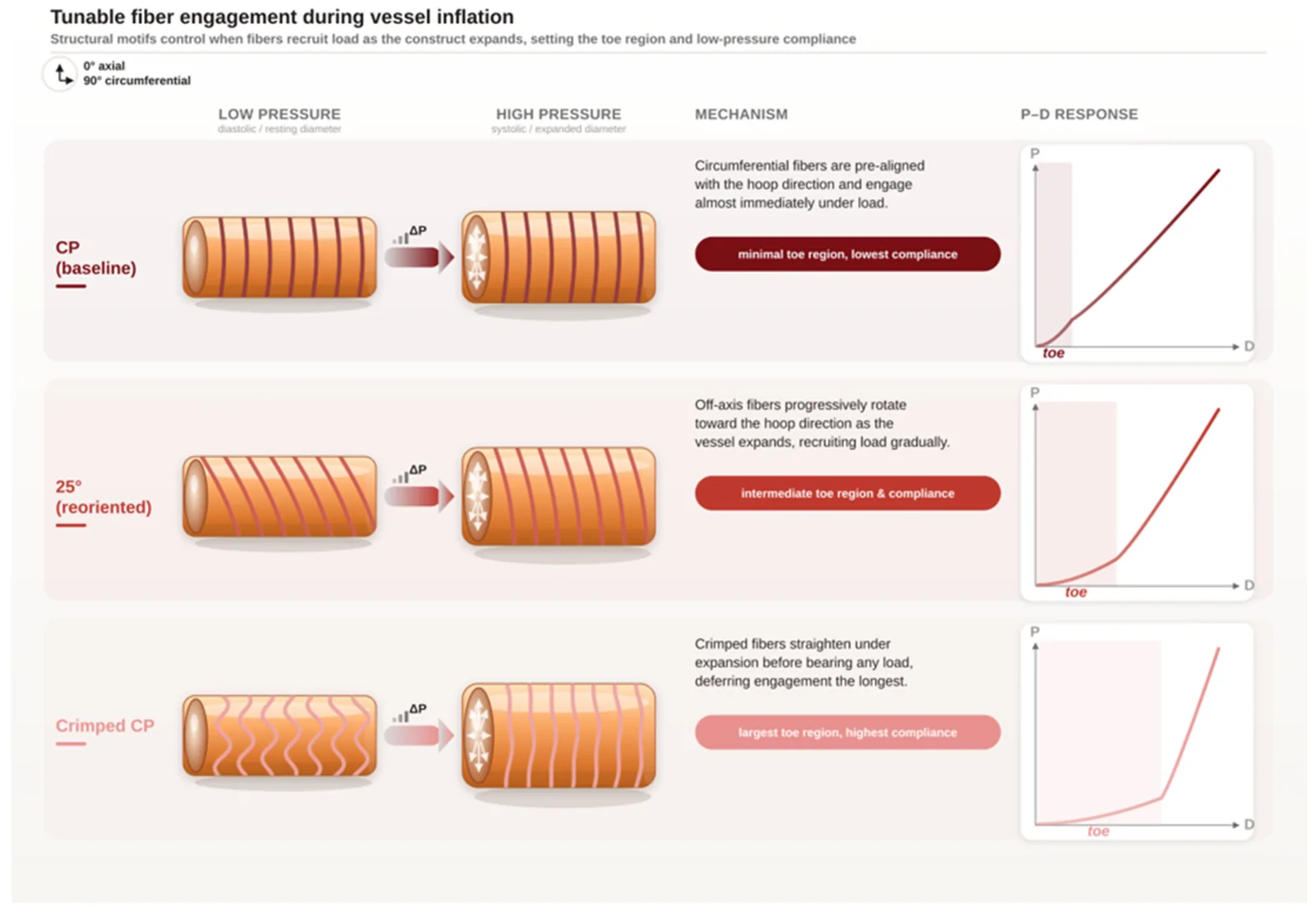
Structural motifs tune fiber engagement during vessel inflation. Schematic of the CP, 25–reoriented, and Crimped CP architectures at low (resting) and high (expanded) pressure, illustrating how each structural motif controls the timing of fiber recruitment during inflation. In the CP architecture, circumferential fibers are pre-aligned with the hoop direction and engage almost immediately, producing a minimal toe region. In the 25° architecture, off-axis fibers progressively rotate toward the circumferential direction as the construct expands, recruiting load more gradually. In the Crimped CP architecture, crimped fibers straighten before bearing load, deferring engagement the longest. The accompanying schematic pressure–diameter (P-D) curves (not to scale) illustrate the resulting increase in toe-region length and low-pressure compliance from CP to 25° to Crimped CP, consistent with the compliance trend reported in Figure 9.

The pressure–diameter and compliance results demonstrate that structural modification of fiber architecture directly governs the pressure-dependent mechanical response of the tubular constructs. In native arteries, nonlinear deformation arises from progressive collagen fiber recruitment, whereby compliant deformation at low pressures is followed by gradual stiffening as fibers become load-bearing [10,11,16,18,20]. This recruitment process is regulated by collagen fiber orientation and crimp [51,54,55,56,57,58]. Consistent with these mechanisms, fiber reorientation and, particularly, crimp increased compliance and pressure-dependent diameter expansion relative to the CP baseline, indicating delayed fiber recruitment during inflation. Importantly, the same recruitment delay was independently reflected in the tensile response, where the toe region progressively extended from CP to 25° to crimped CP, mirroring the architectural trends observed under internal pressurization. This structural mechanism of pressure-dependent fiber engagement is illustrated schematically in Figure 10.

Rather than representing a single optimal mechanical state, native arteries span a physiological spectrum in which structural motifs, including fiber orientation, crimp, and fiber recruitment, regulate pressure-dependent deformation according to age and vascular condition. Young arteries achieve compliant behavior through delayed fiber recruitment, whereas aging progressively reduces crimp, advances fiber recruitment, and shifts the mechanical response toward earlier stiffening [14,15]. By sequentially introducing these same structural motifs into a simplified synthetic architecture, the present platform reproduced a corresponding progression in mechanical behavior, from the stiffer CP configuration toward the more compliant crimped architecture. These findings demonstrate that vascular structural motifs can function as programmable engineering design parameters, enabling a single material platform to reproduce distinct physiological mechanical phenotypes through architecture alone.

Comparison with reported coronary artery behavior [12,13,46] further demonstrates that structural architecture systematically modulates physiological mechanical response. The three architectures progressively shifted along the aged-to-young mechanical continuum, as reflected by pressure-dependent compliance, the Native Similarity Score, and statistical comparison with physiological reference data. While the crimped CP architecture most closely approached the young phenotype, none of the investigated architectures fully reproduced either physiological target, indicating that additional structural motifs may further refine biomimetic mechanical behavior.

Importantly, these architecture-dependent changes were achieved without significant differences in elastic modulus, while compliance and toughness increased progressively with fiber reorientation and crimp. This demonstrates that relatively simple modifications in fiber organization can systematically tune vascular mechanical behavior without the conventional stiffness–compliance trade-off associated with fiber-reinforced composites.

The results suggest that vascular graft design may benefit from tunable rather than universally maximized compliance behavior. Native arterial mechanics vary considerably between individuals and are strongly influenced by age and disease. Younger arteries typically exhibit higher compliance and delayed stiffening behavior, whereas aged arteries demonstrate reduced compliance and earlier fiber recruitment. In this context, the ability to tailor the mechanical response of the graft through structural modification may provide a strategy for improving mechanical compatibility with different physiological conditions. Rather than targeting a single fixed compliance value, the platform can, in principle, be directed toward young or aged coronary behavior as a deliberate design choice by selecting the fiber architecture that positions the construct at the intended point along the aged-to-young continuum. This reframes compliance matching as a patient- and application-specific design target rather than a single universal benchmark to be maximized.

The present results demonstrate that macro-scale structural motifs alone provide a powerful means of tuning vascular mechanics while preserving construct stiffness. Beyond the architectural strategies investigated here, additional hierarchical structural motifs at smaller length scales may provide complementary opportunities for further expanding the programmable mechanical design space. In other fibrous systems, nanoscale crimping and micro/nano hierarchical organization have been shown to influence not only toe-region mechanics but also fracture and tear resistance [35,51], suggesting that integrating hierarchical structural features with the macro-scale architectural control demonstrated here may further enhance both compliance and damage tolerance. Nevertheless, the present architectures already define a tunable mechanical design space in which compliance and toughness can be adjusted largely independently of stiffness.

Unlike most reported small-diameter vascular grafts, which are primarily benchmarked using burst pressure and suture retention [24,25], the present study emphasizes the complete pressure-dependent compliance curve and its similarity to native coronary mechanics. This complementary perspective directly addresses the compliance mismatch mechanism underlying graft failure and provides a more physiologically relevant framework for evaluating vascular graft mechanics.

The present study establishes a biomimetic design framework in which vascular structural motifs serve as programmable engineering parameters for controlling pressure-dependent mechanics.

Several limitations of the present study should be acknowledged. First, this work focused specifically on the mechanical design of the constructs—namely how biomimetic structural motifs govern the tensile and pressure-dependent response—and did not include biological characterization. Accordingly, the biocompatibility and cellular response of the constructs were not directly assessed here. It should be noted, however, that the individual constituents of the present system are well established as biocompatible: silk fibroin is a widely used, FDA-approved biomaterial with demonstrated biocompatibility [59], and the tough alginate–polyacrylamide IPN matrix used here has been shown to be biocompatible in both in vitro and in vivo settings [44]. The biological suitability of the combined silk fiber-reinforced IPN system for cardiovascular applications was likewise supported in our previous work [38]. Second, biological performance under physiologically relevant conditions—including endothelialization, host cell response, and long-term functional remodeling—was outside the scope of the present study and remains to be evaluated. Third, the mechanical evaluation was performed under quasi-static conditions; dynamic, pulsatile, and long-term fatigue behavior were not investigated. While the crimp wavelength was measured directly in both the bare and hydrated-embedded states, the out-of-plane crimp amplitude was not directly measured from the current imaging plane and corresponds to the nominal mold-defined value; direct side-view measurement of the as-built crimp amplitude represents a useful refinement for future work. In addition, although a dedicated thermally treated non-crimped control group was not included, the thermal fixation used to impose crimp is not expected to account for the observed mechanical differences (see Section 2.2); nonetheless, a matched thermal control represents a useful confirmation for future work. Future work will therefore extend this architecture-driven framework toward direct biological validation, dynamic mechanical testing, and in vivo assessment, building on the mechanical design principles established here.

## 5. Conclusions

This study demonstrates that discrete vascular structural motifs, specifically fiber reorientation and crimp, can function as programmable engineering parameters for tuning the pressure-dependent mechanical behavior of fiber-reinforced hydrogel constructs without altering material composition or compromising construct stiffness. Sequential incorporation of these motifs systematically shifted the mechanical response along the aged-to-young coronary continuum while maintaining physiological pressure-dependent behavior within the reported variability of native coronary arteries. More broadly, these findings establish structural architecture as an independent design axis for engineering vascular mechanics, supporting biomimetic design strategies in which mechanical function is programmed through architecture rather than material composition alone.

## Figures and Tables

**Table 1:** Geometry of the tubular constructs used for pressure–diameter and compliance testing.

Material System	*n*	Inner Diameter (mm)	Wall Thickness (mm)
CP	4	4.99 ± 0.41	1.35 ± 0.04
25°	4	4.93 ± 0.56	1.31 ± 0.05
Crimped CP	4	4.83 ± 0.45	1.36 ± 0.03

Values are mean ± SD; *n* = number of samples per group.

**Table 2:** Geometry of the fabricated silk fiber-reinforced laminate samples for each architecture.

Material System	*n*	Thickness (mm)	Width (mm)	Length (mm)	Fiber Volume Fraction (VF)
CP	6	1.4 ± 0.1	4.9 ± 0.7	8.3 ± 1.0	0.45 ± 0.02
25°	6	1.4 ± 0.1	4.8 ± 0.9	8.5 ± 0.9	0.46 ± 0.02
Crimped CP	6	1.4 ± 0.1	4.9 ± 0.5	8.5 ± 1.7	0.46 ± 0.01

Values are mean ± SD; *n* = number of samples per group.

## Data Availability

The data supporting the findings of this study are available from the corresponding author upon reasonable request.

## Abbreviations

The following abbreviations are used in this manuscript:

Alg-PAAm	Alginate–polyacrylamide
ANOVA	Analysis of variance
APS	Ammonium persulfate
CP	Cross-plied
Dacron	Polyethylene terephthalate
DDW	Double-distilled water
*D/D*_50_	Diameter normalized to the diameter at 50 mmHg
ePTFE	Expanded polytetrafluoroethylene
IPN	Interpenetrating polymer network
MBAA	N,N′-methylenebisacrylamide
P-D	Pressure–diameter
TEMED	N,N,N′,N′-tetramethylethylenediamine
UTS	Ultimate tensile strength
FVF	Fiber volume fraction
